# Advanced transverse colon cancer highly indicative of invasion to the duodenum and pancreas

**DOI:** 10.1002/ccr3.2447

**Published:** 2019-10-06

**Authors:** Takayuki Yamada, Ichiro Sakamoto, Susumu Ohwada

**Affiliations:** ^1^ Asunaro Clinic Takasaki Japan; ^2^ National Hospital Organization Takasaki General Medical Center Takasaki Japan; ^3^ Askohwada Medical Consultant Office Maebashi City Gunma Prefecture Japan

**Keywords:** direct invasion to the duodenum and pancreas, locally advanced right‐sided colon cancer, pancreaticoduodenectomy, right hemicolectomy

## Abstract

Our images showed an advanced transverse colon cancer highly indicative of an invasion to the duodenum and pancreas. For en bloc tumor resection, surgeons should make a deliberate operative plan.

## Graphical abstract

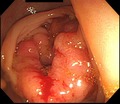

What diagnosis could be made from the endoscopic image and abdominal radiograph, and how should the condition be managed?

A 68‐year‐old man presented with periodic abdominal pain, prolonged diarrhea for 3 months, and weight loss of 3 kg. A colonoscopy image revealed fully circumferential transverse colon tumor stenosis (Figure 1), and a postcolonoscopy abdominal radiogram showed an apple core sign (Figure 2, arrow), indicating albescent large bowel obstruction due to transverse colon cancer. Biopsy revealed a moderately differentiated adenocarcinoma. An esophagogastroduodenoscopy image showed a submucosal tumor of the duodenal bulbus (Figure 3), indicating direct tumor invasion. The computed tomography findings were highly indicative of duodenal and pancreatic head invasion of the transverse colon cancer (Figure 4). We planned en bloc pancreaticoduodenectomy and right hemicolectomy for the locally advanced right‐sided colon cancer preoperatively. On laparotomy, the tumor showed neither lymph node metastasis nor duodenal or pancreatic invasion. Thus, a radical right hemicolectomy alone was performed curatively.^1^, ^2^ Locally advanced right‐sided colon cancer may directly invade the duodenum or pancreatic head. Our images showed an advanced transverse colon cancer highly indicative of an invasion to the duodenum and pancreas. For en bloc tumor resection, surgeons should make a deliberate operative plan.^1^, ^2^

**Figure 1:**
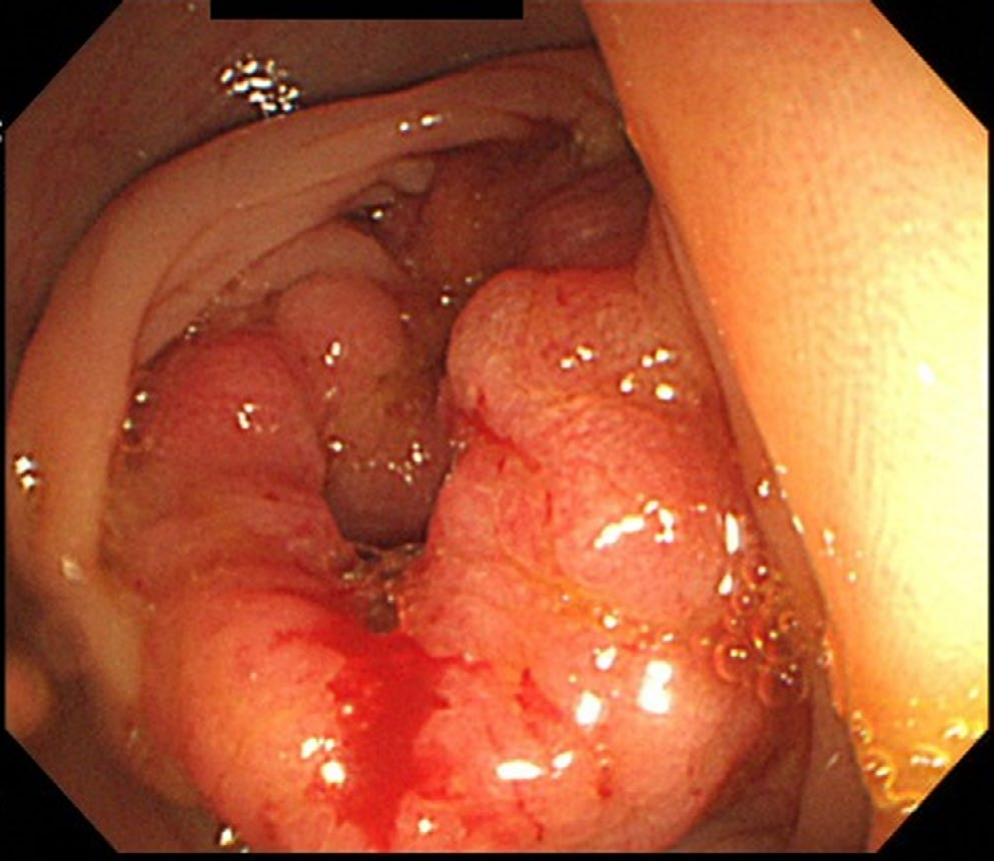
A colonoscopy image revealing fully circumferential transverse colon tumor stenosis

**Figure 2:**
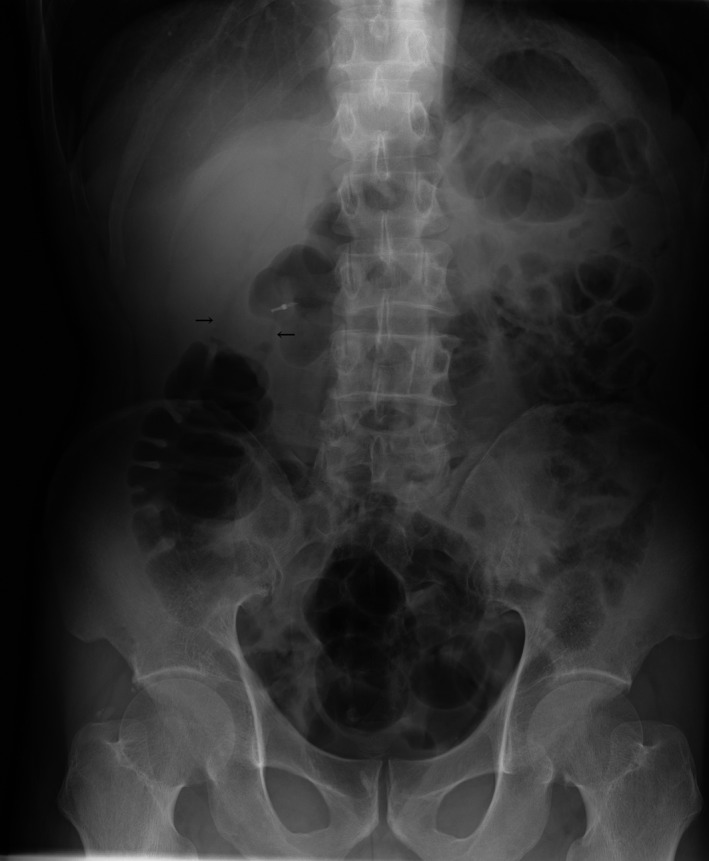
A postcolonoscopy abdominal radiogram showing an apple core sign (arrow)

**Figure 3:**
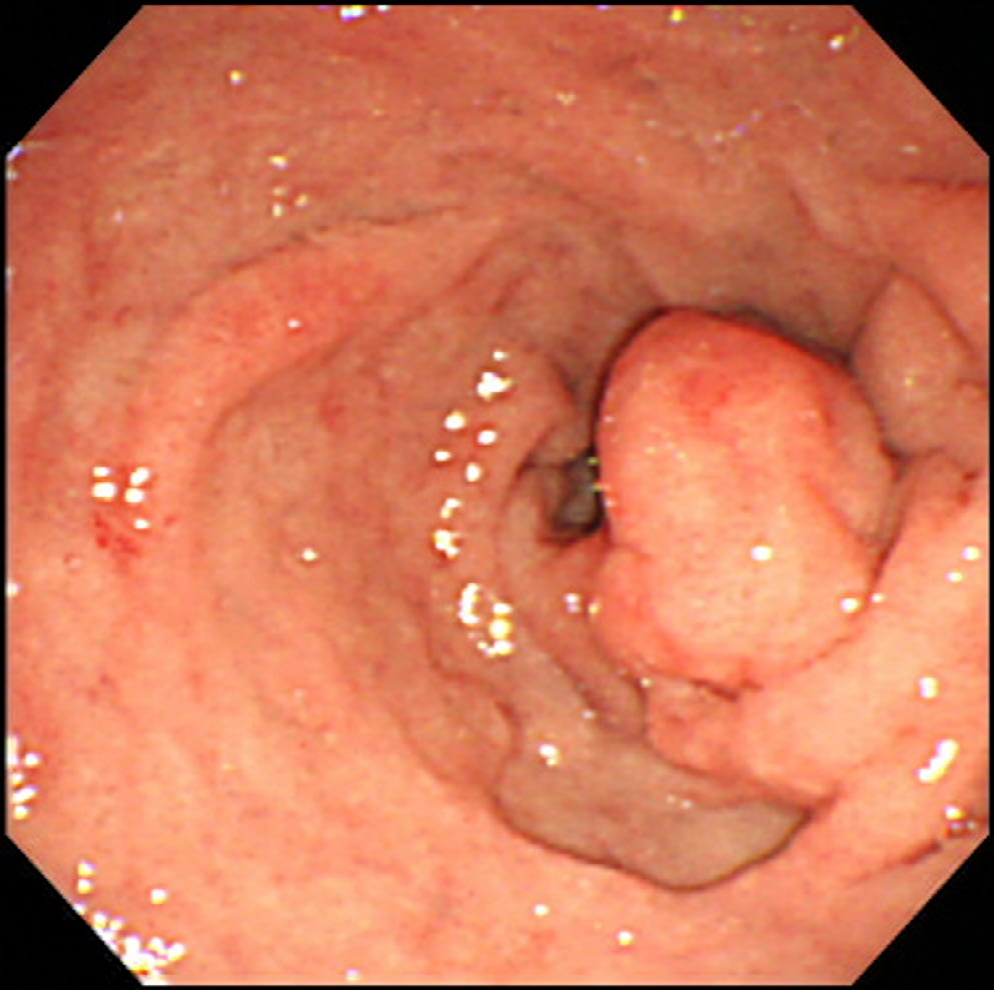
An esophagogastroduodenoscopy image showing a submucosal tumor of the duodenal bulbus

**Figure 4:**
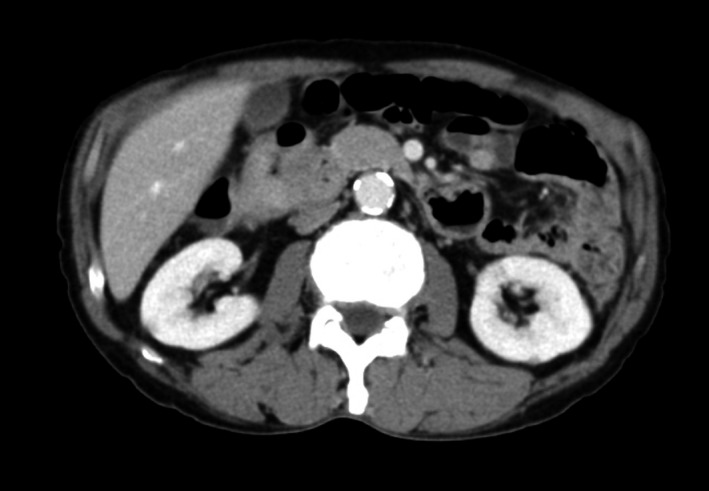
The computed tomography findings were highly indicative of duodenal and pancreatic head invasion of the transverse colon cancer

## AUTHOR CONTRIBUTIONS

TY: Served as a diagnostician and first author. IS: Served as an operator.SO: Served as a supervisory doctor.
